# Dark triad personality traits prediction with AI

**DOI:** 10.1192/j.eurpsy.2021.386

**Published:** 2021-08-13

**Authors:** A. Mereu

**Affiliations:** Research performed independently, Cagliari, Italy

**Keywords:** Artificial Intelligence, Personality, traits, psychometry

## Abstract

**Introduction:**

The dark triad is composed by the personality traits Machiavellianism, narcissism and psychopathy (MNP). Their complexity can make them difficult to interrelate. Artificial intelligence (AI) could help in this endeavour.

**Objectives:**

To investigate whether AI could predict MNP from themselves.

**Methods:**

Data from 210 questionnaires were analysed using an AI. The short Dark Triad questionnaire (SD3) was used to assess MNP. Two of the MNP scores were employed to predict the third one and the procedure was repeated for all of them alternatively. The AI was conservatively tuned to maximize the one-way random intraclass correlation coefficient (ICC) between predicted and real values. Pearson’s r was calculated too. The free and open source programming language R was used for all the analyses. Dataset source: Borráz-León, Javier I. (2020), “Dark triad, attractiveness, mate value, and sexual partners”, Mendeley Data, V1, doi: 10.17632/87vx6jfnrp.1

**Results:**

Machiavellianism, narcissism and psychopathy predictions obtained ICC of 0.593, 0.335, 0.505 and Pearson’s r of 0.608, 0.346, 0.548 respectively. The results were indicative of fair performance, mainly for Machiavellianism and psychopathy.

**Conclusions:**

AI might be useful to predict MNP. This could be utile in many situations, such as dealing with missing data or deciding whether to formally test someone. Finally, the AI used in this study is freely available, allowing anyone to experiment.

**Disclosure:**

No significant relationships.

